# Effect of Polyvinylidene Fluoride Membrane Production Conditions on Its Structure and Performance Characteristics

**DOI:** 10.3390/polym14235283

**Published:** 2022-12-03

**Authors:** Sergey Fomin, Evgenia Shirokova, Iren Kraeva, Ivan Tolstobrov, Andrey Bushuev, Kirill Yuzhanin, Boris Ananchenko, Alexandre A. Vetcher, Alexey Iordanskii

**Affiliations:** 1 Institute of Chemistry and Ecology, Vyatka State University, 36 Moskovskaya St., 610000 Kirov, Russia; 2 Institute of Biochemical Technology and Nanotechnology (IBTN), Peoples’ Friendship University of Russia (RUDN), 6 Miklukho-Maklaya St., 117198 Moscow, Russia; 3 Complementary and Integrative Health Clinic of Dr. Shishonin, 5 Yasnogorskaya St., 117588 Moscow, Russia; 4 N.N. Semenov Federal Research Center for Chemical Physics, Russian Academy of Sciences, 4 Kosygin St., 119334 Moscow, Russia

**Keywords:** PVDF membrane, freeze-casting, nucleation, crystallization

## Abstract

Poly (vinylidene fluoride) membranes were prepared by freeze-casting. The effects of PVDF concentration, and freezing temperature on the morphology, crystallization, and performance of prepared membranes were examined. Polymer concentration was varied from 10 to 25 wt%. The freezing temperature was varied from −5 to −25 °C. Dimethyl sulfoxide (DMSO) and distilled water were used as solvents and non-solvents, respectively. The first step of this study was devoted to estimating the optimal concentration of PVDF solution in DMSO. Membranes prepared at different ratios were characterized using physical and mechanical characteristics and porosity. The second step was to optimize the time required for the production of the membranes. In the third step, it was shown that the freezing temperature had a remarkable effect on the morphology of the membranes: as the temperature decreases, there is a transition from spherulite structures to interconnected pores. It was shown that the diversity in the pore pattern for PVDF affects remarkably the water permeability through the polymer membrane. During the monitoring of the spread of crystallized areas during the formation of the membrane, it was found that the crystallization of the solvent begins at localized points of the microscale, further crystallized areas spread radially or unevenly along the surface of the solution, forming contact borders, which can lead to changes in the properties of the membrane in its area.

## 1. Introduction

Two of the 17 UN global goals are directly related to the provision of clean drinking water and the rational use of water resources by the world’s population [1]. Therefore, the development of water purifying technologies and the reduction in cost and energy consumption during filtration are actual and practically urgent goals of polymer science.

Membrane technologies (micro-, ultra-, and nanofiltration, membrane distillation, and reverse osmosis) are widely used in the petrochemical, pharmaceutical, food, and other industries [2,3]. One of the extensively used membrane materials designated for micro- and ultrafiltration is polyvinylidene fluoride (PVDF) [4,5] which possesses a thermoplastic behavior, high thermal and mechanical stability performance, excellent chemical resistance, UV resistance, and electroactive properties. Among all fluoroplastics, it ranks in second position in terms of production volume [6,7,8,9,10,11]. Selective solubility of PVDF in such common solvents as N-methyl-2-pyrrolidone (NMP), N-dimethylformamide (DMF), dimethyl sulfoxide (DMSO), and N,N-dimethylacetamide (DMAc) [12] makes it possible to obtain membranes by the traditional phase inversion in solutions, namely thermally-induced phase separation (TIPS) and non-solvent-induced phase separation (NIPS) [13,14,15,16,17,18,19,20,21,22].

Another method of producing porous materials that have attracted certain attention in the last two decades is freeze-casting, also known as ice-templating [23,24,25]. In this case, the porous material is formed by freezing a system comprising a target component and a freezing liquid. Homogeneous or directed freezing of the system is ensured by isotropic or anisotropic cooling, which forms porous structures of various architectures. Next, the frozen liquid is removed (the method of removal depends on the type of fluid used), and the formed porous structure is preserved and mimics the morphology of the previously frozen liquid.

This method speaks to the possibility of producing porous structures while retaining the flexibility of the freezing process: the structure of the pores can be adjusted by changing the characteristics of the system, as well as the freezing conditions.

A similar technique has been used to produce membranes/porous materials from PVDF. Thus, the effect of the PVDF concentration in the solution on the properties of the membranes produced has been evaluated [26], while the freezing temperature remains stable (−10 °C), and the freezing lasts 2 hours. It had been found that the mechanical properties of the membranes strengthen, while the porosity and water permeability of the membranes gradually decreases with the increase in PVDF concentration in the solution.

In [27] the effect of the base plate’s material (glass, aluminum) and the cooling rate up to −30 °C, which is determined by the plate’s thermal conductivity, on some 20 wt% PVDF solution, have been studied. The resulting membranes have pores up to 30 nm and demonstrated superior water permeability compared to traditional NIPS and TIPS membranes with comparable pore sizes.

The studies on the membranes, prepared from PVDF solutions of varying molecular weights of 15 wt% concentration, were reported in [28]. There it had been attempted to register the effect of freezing temperature on the structure and properties of the membranes. It has been concluded that the increase in the cooling temperature results in an increase in the pore size and membrane water permeability.

In the articles mentioned above, DMSO, which has a low melting point, is non-toxic, and is recognized as a «green» solvent, has been used as a freezing liquid [29,30].

However, as rightly noted in [25], each study presents, to some extent, its own set of parameters, so it is almost impossible to conclude any one article. Moreover, there are few publications on the freeze-casting of PVDF membranes, so a systematic review of the correlation between the processing, structure, and properties of the produced porous materials is very difficult.

This paper has attempted to assess the effect of solution concentration and freezing temperature on the structure and operational characteristics of the produced membranes. For the first time, Russian-made PVDF, differing in its molecular weight characteristics from the ones previously used, has been taken as the object of the study.

## 2. Materials and Methods

In this research we used F2M-E polyvinylidene fluoride (PVDF) (HaloPolymer, LLC, Kirovo-Chepetsk, Russia) as a white powder (melt flow index is 3.0–8.0 g/10 min); dimethyl sulfoxide (DMSO) (Vecton, JSC, Moscow, Russia) as reagent grade, which is a solvent of hazard class 4 (melting point 18.55 °C).

**Figure 1:**
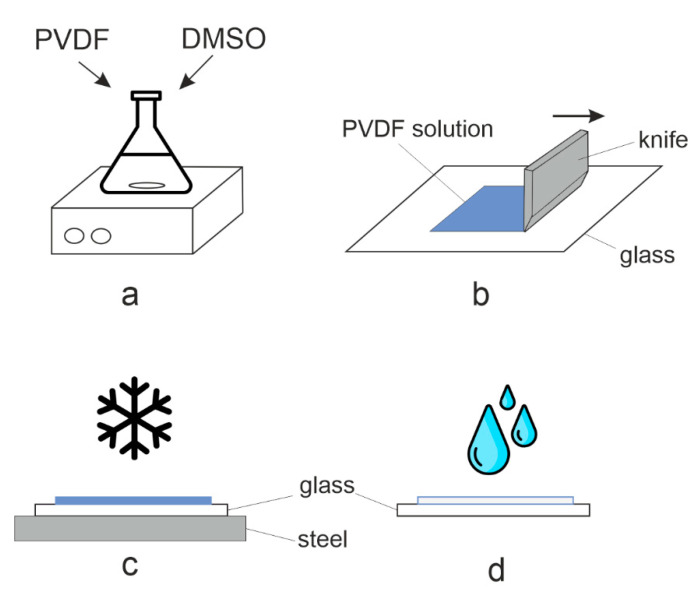
Steps of PVDF membranes production by freezing ((**a**–**d**) detailed explanation are in the text).

The sequence PVDF membranes production by freeze-casting is shown in Figure 1.

We mixed PVDF powder and DMSO for 2 h in a heat-resistant beaker at 60 °C using an ES-6120 magnetic stirrer to obtain a solution of a predetermined concentration. The resulting polymer solution was cooled to (23 ± 2) °C (Figure 1a).

The polymer solution was distributed on 0.5 cm thick glass plate (in the first series of experiments the distributed predetermined 500 μm-thick solution and in subsequent series −200 μm-thick) (Figure 1b).

Later on, the glass plate carrying the PVDF solution was placed in a Pozis Paracels freezer (Pozis, JSC, Zelenodolsk, Russia) onto a pre-cooled 1 cm thick metal plate to ensure better thermal conductivity and cooling rate (Figure 1c). After the specified time, the substrate was transferred into distilled water, the membrane was separated from the substrate and kept until the solvent was completely removed (Figure 1d). The resulting membranes were stored in distilled water.

Concentrations of the tested solutions, the inside-the-freezer temperature, the freezing duration are presented in Table 1.

Once the time is up, the glass plate was removed from the freezer and immersed into distilled water at 2–4 °C. The produced membranes were washed with distilled water until the solvent was completely removed and then stored in distilled water at (23 ± 2) °C (Figure 1d).

To visually record the process of membrane formation (the DMSO crystallization), an Espada U1600× digital microscope (ESPADA System Corp., Beijing, China) was installed in the freezer, the image has been output onto a laptop.

The following parameters of the produced membrane were evaluated:

Porosity (P) is defined as the ratio of the pore volume to the total membrane volume. Moisture from the surface of the samples has been preliminarily removed with filter paper, then the samples were subjected to drying for 8 h at 75 ± 2 °C (it was found by gravimetric analysis that this time is sufficient for the removal of moisture completely). The porosity was determined by the formula:

(1) $$P\left(\%\right)=\frac{(W_{wet}-W_{dry})D_{W}}{(W_{wet}-W_{dry})/D_{W}+W_{dry}/D_{F}}\cdot 100$$

where *P*—porosity, %;

$W_{wet}$ and $W_{dry}$—the weight of the wet and dry membrane, respectively;

$D_{W}$ and $D_{F}$—the water and PVDF densities, respectively.

The 30 mm × 10 mm membrane samples have been used to assess physical and mechanical properties (tensile strength). A Shimadzu AG-X5 tensile machine (Shimadzu Corp., Kyoto, Japan) was used in five iterations at the grip’s speed of 3 mm/min at (23 ± 2) °C.

**Figure 2:**
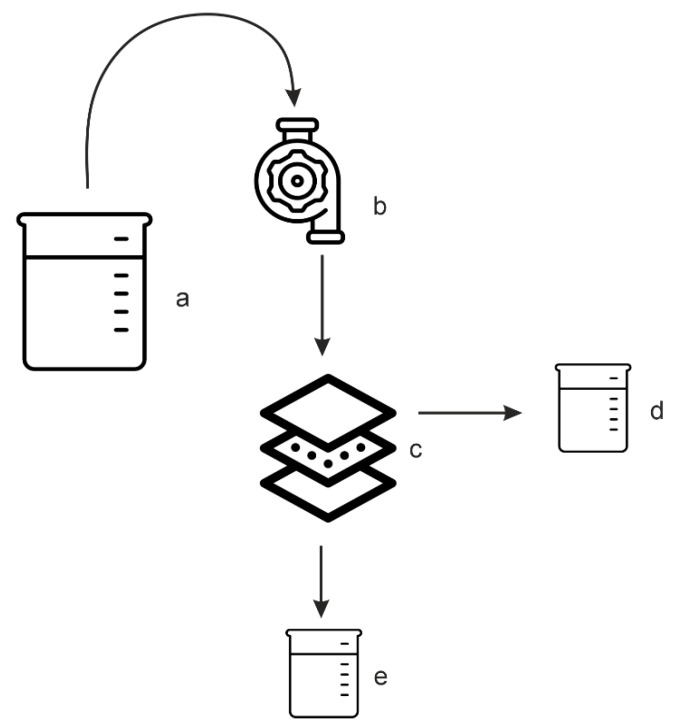
Schematic of the unit for assessing a membrane’s water permeability: (**a**)—water intake collector; (**b**)—water pump; (**c**)—filtration cell; (**d**)—retentate collector; (**e**)—permeate collector.

The performance of the membrane has been evaluated by its permeability to distilled water. To assess the membrane permeability, a special unit was used (its schematic diagram is shown in Figure 2).

The study used a 2 cm × 4 cm cell, with the permeation area of 8 × 10^−4^ m^2^. The initial flow of distilled water through the membrane was 27.0 mL/min; pressure 0.1 bar. The volume of water passing through the membrane per 30 min was recorded. The tests were run in triplicate. The membranes’ water permeability was calculated by the formula:

(2) $$J_{wtr}=\frac{V}{A\cdot \Delta t},$$

where *J*_wtr_—the water flux through the membrane, l/(m^2^ × h);

*V*—volume of filtered water, l;

*A*—8 × 10^−4^ m^2^ membrane area;

Δ*t*—filtration time, h.

The thermal properties of membranes prepared at different freezing temperature were characterized by differential scanning calorimetry (DSC) using DSC-60 calorimeter (Shimadzu Corp., Kyoto, Japan). The samples were heated at 10 °C/min at 40 to 200 °C. The results were used to estimate melting enthalpy ($H_{m}$) and crystallinity degree ($\chi_{c}$). The following equation was used to calculate $\chi_{c}$:

(3) $$\chi_{C}=\frac{H_{m}}{H_{m}^{*}}\cdot 100\%,$$

where $H_{m}$—the melting enthalpy calculated from the melting peak of the DSC curve, J/g;

$H_{m}^{*}$—the melting enthalpy of totally crystallized PVDF, J/g.

The crystalline structure of membranes was determined using an X-ray diffractometer XRD-7000 (Shimadzu Corp., Kyoto, Japan) with a graphite monochromator and Cu-Kα radiation (1.54 Å). The applied voltage and current of the X-ray tube were 40 kV and 30 mA, respectively. The XRD measurements were performed in the transmission geometry at 2θ 5°–50° (scanning speed of 0.02° per second).

The morphology of the membrane samples was studied by scanning electron microscopy (SEM) in slow secondary electrons mode at an accelerating voltage of 10 kV with a JEOL JSM-6510 LV microscope (Shimadzu Corp., Kyoto, Japan). The samples were dried to remove water from inside the pores and then crushed in liquid nitrogen. The prepared samples were later coated with 10 nm thick gold. The surface of the membranes (separation surface—the surface contacting the glass and metal plates) and its cross-section were examined.

To assess the size of the pores formed, we used ImageJ (public domain scientific image processing and analysis software, licensed as Open Source Software (OSS), https://imagej.net/software/imagej/ (accessed on 20 November 2022)) and editing software to analyze the SEM images. The pore size (Feret’s diameter) (FD) was assessed in the separation layer (a layer formed near the contact area with the glass and the metal plates) as well as on the separation surface. It should be underlined that the registered pore sizes will be used in the current study as a reference value. We do not compare these data to the pore sizes registered by traditional porosimetry methods, such as the bubble-point method, mercury intrusion porosimetry, and the permeation measurement, which are traditionally used to evaluate pore sizes [31]. Each of these methods has its limitations.

The bubble-point method is suitable for determining the largest pores. The major disadvantage is different results with different fluids.

The mercury intrusion porosimetry allows you to determine both the pore size and the pore size distribution. The list of disadvantages includes caution when working with mercury; the possibility of damaging the membrane at high pressures; and counting dead-end pores.

The permeation measurement is simple to perform, but requires some certainty of the real structure of the pores. Therefore, in our case, there will be difficulties in data interpretation.

From the abovementioned points, using SEM for pore size estimation is appropriate due to the simplicity of sample preparation and the possibility of automating data processing using software such as ImageJ.

## 3. Results

### 3.1. Selection of the Optimal Concentration of PVDF-DMSO Solution

**Figure 3:**
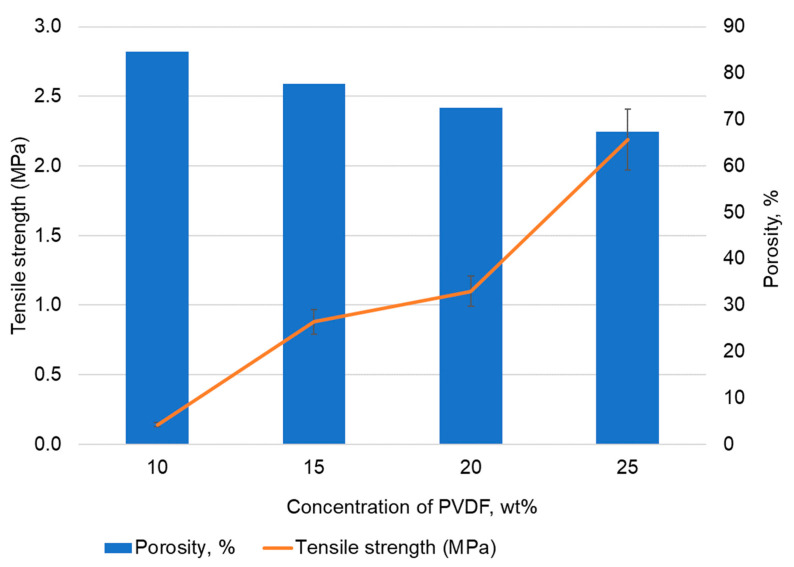
Comparison of the tensile strength and porosity of the prepared membranes.

In the first step, we determined the concentration of PVDF-DMSO solution to ensure an optimal combination of production and performance properties. To do this we prepared membranes from solutions of concentrations of 10, 15, 20, and 25 wt%. Their porosity and physical and tensile strength are presented in Figure 3. For this series of experiments, we kept the samples in the freezer for a long time (120 min) to ensure complete solvent crystallization within the entire sample volume.

As the data presented in Figure 3 demonstrate, an increase in the solution concentration results, predictably enough, in a decrease in the membranes’ porosity; the maximum stress at rupture increases from 0.14 MPa for 10 wt% solution concentration membranes to 2.19 MPa for 25 wt% solution concentration membranes. As the concentration of the solution increases, the porosity of the prepared membranes decreases, while the tensile strength increases.

The 10 wt% solution has a low viscosity, which greatly facilitates the process of membrane production by casting the solution. However, the membranes have demonstrated insufficient strength and cracks on their surface, which hinder any further work.

It is known that the mechanical properties of porous materials are mainly determined by volume porosity [15]. It is expected that membranes obtained from a solution of 30 wt% concentration will have even greater tensile strength. However, as the concentration of PVDF increases, the viscosity of the solution increases significantly. It was shown in [26] that an increase in the concentration of PVDF in DMSO from 20 to 30% leads to an increase in the viscosity of the solution by almost 8 times. The high viscosity of the solution hinders the process of membrane formation. Thus, with an increase in the concentration of solutions, both an increase in the viscosity of the solutions and an increase in the strength properties of membranes occur. A solution with a concentration of 25 wt% has a viscosity level that makes it possible to obtain a high-quality membrane surface without technological problems. At the same time, membranes obtained from a solution of 25 wt% concentration have higher strength properties than those formed from solutions with a lower concentration. Thus, a concentration of 25 wt% is optimal.

The tensile stress for 25 wt% solution concentration membrane is higher than those of thermally induced phase separation PVDF membranes using dibutyl phthalate as the solvent (1.79 ± 0.14 MPa) [32] and comparable to those of NIPS method membrane using dimethylacetamide (2.2 ± 0.16 MPa) as the solvent [33]. However, this value is less than those of vapor-induced phase separation PVDF membranes (3.24 ± 1.1 MPa) [34] and thermally induced phase separation ones using a mixture of dibutyl phthalate/dioctyl phthalate solvents (4.2 ± 0.1 MPa) [35].

Based on the obtained data, we can conclude that the optimal concentration of F2M-E PVDF (HaloPolymer, OJSC; Moscow, Russia) solution in DMSO is 25 wt% since the produced membranes have acceptable strength and better technological properties as compared to lower concentration solutions membranes.

The need to select a PVDF solution concentration in DMSO that could enable the optimal combination of technological and operational properties is described in [36] by the authors. The use of Solef 1008 PVDF (Belgium) of a lower molecular weight is described in the article. Due to its lower molecular weight, the optimal concentration is higher and amounts to 30 wt%.

### 3.2. Optimization of Membranes Production Time

The second step was to optimize the time required for the production of the membranes. The minimum time in the freezer was determined through the use of photo and video fixation means. Such technique ensures the completion of the DMSO crystallization process in the sample. The DMSO crystallization process at −10 °C was found to take 260 s. In further experiments, the exposure time was tripled (to 15 min) to ensure the complete solvent crystallization process throughout the sample volume.

**Figure 4:**
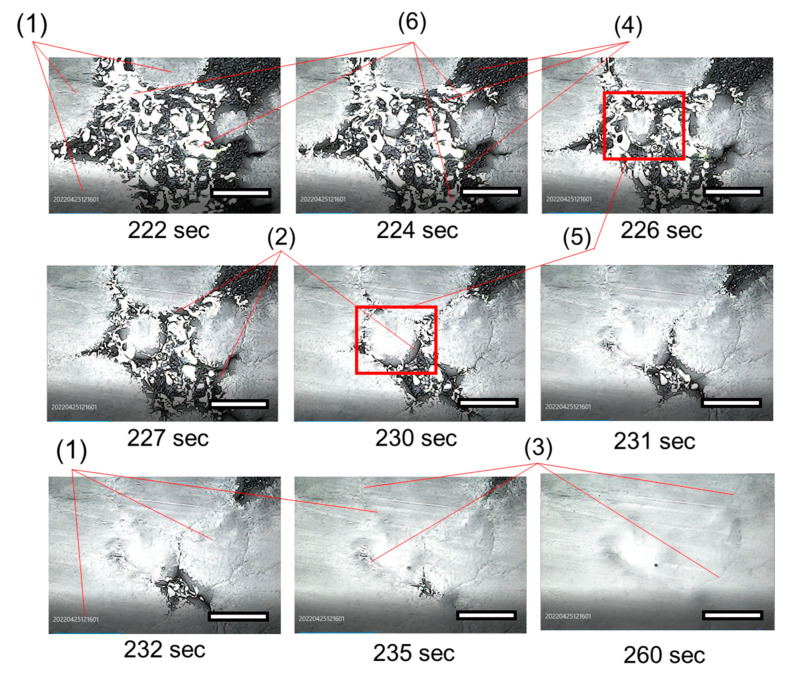
Photo image of growing crystallized areas during the membrane formation at −10 °C (the length of the scale bar (horizontal) 5mm): (**1**)—crystallized areas, (**2**)—transitional areas, (**3**)—contact borders, (**4**)—PVDF solution, (**5**)—growing crystallized area (red square), (**6**)—glare from a light source.

Additionally, at a temperature of −10 °C, at the macrolevel, we carried out observations of the spread of crystallized areas in time during the formation of the membrane. A video of this process is provided in Supplementary Video S1. Images from the microscope camera taken during the observation of DMSO crystallization and membrane formation are shown in Figure 4.

The central part of the pictures has glare on the surface of the solution from the light source (LED lamp). It should be noted that these reflections were difficult to avoid due to reflection from the smooth surface of the solution. On the other hand, the crystallized areas (1) are devoid of reflections, which makes it possible to visualize well the growth of the crystalline phase.

The images obtained at different times clearly show the growth of a crystallized area in the center of the survey site. This area appears at a time of 224 s and then spreads almost radially, touching other crystallized areas, also spreading on the surface, but from the periphery of the shooting area. The images also clearly show “transitional” areas (2), which look like dark framing bands along the growth front of the crystallized areas. Until the end of the observation on the surface of the membranes under a microscope, the contact borders of growing crystalline formations are quite clearly visible (3). These contact borders are maintained throughout the experiment.

From the observations made, the following conclusions can be drawn regarding the specifics of the formation of membranes by the freeze-casting method:

- crystallization of the solvent, which is in a metastable state, does not begin over the entire surface on which the solution is applied, but only at individual points of the microscale, probably by the mechanism of heterogeneous nucleation; further crystallized areas spread radially and unevenly over the surface of the solution;
- the growth rate of crystallized areas is well fixed by visual observation in an optical digital microscope;
- in the process of solvent crystallization, at least four areas can be distinguished on the surface of the formed membrane: completely crystallized (1), transitional areas belonging to the moving growth front of crystalline areas (2), contact borders of crystalline areas (3), non-crystallized polymer solution (4);
- crystallized areas reach macroscopic dimensions of the order of 10–20 mm.

### 3.3. Establishing the Effect of Freezing Temperature

The third step’s objective has been to establish the effect of freezing temperature on the structure and performance of the membranes produced. We have produced membranes from a 25 wt% PVDF-DMSO solution at −5, −10, −15, −20, and −25 °C. The produced membranes have been evaluated for thickness, porosity, physical and mechanical characteristics, thermal properties, and water permeability. The key features of the produced membranes are presented in Table 2.

**Figure 5:**
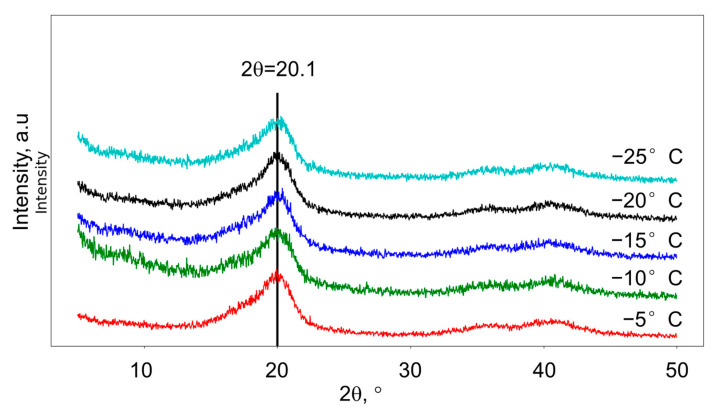
XRD spectra of membranes produced at different freezing temperature.

XRD spectra of produced membrane are shown in Figure 5.

**Figure 6:**
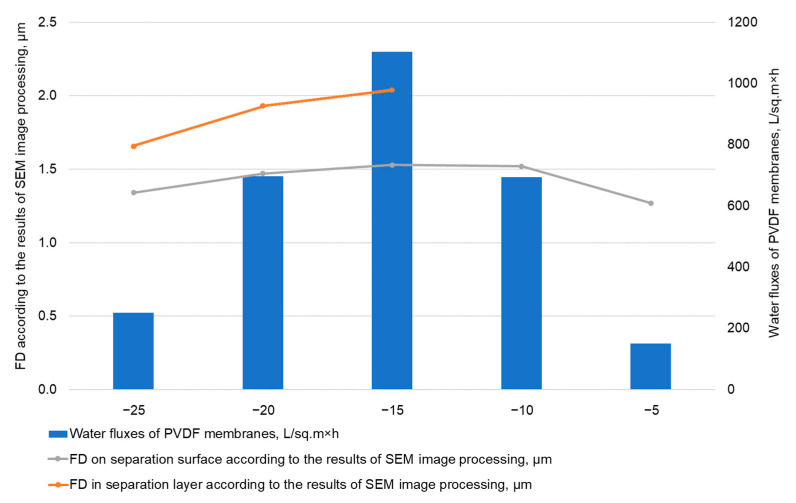
Water fluxes and FD according to the results of SEM image processing for the produced membranes.

The membranes’ water permeability values and FD according to the results of SEM image processing with ImageJ are shown in Figure 6.

Having studied the produced membranes, we have found that the solution freezing temperature affects the properties of the membranes. The decrease in the freezing temperature leads to the increase in the membranes’ thickness from 135 to 180 μm. The porosity decreases slightly, while the water permeability changes along a curve with a maximum at −15 °C.

XDR and DSC results showed that PVDF membranes prepared at different freeze temperature consist of mainly β-phase (there were small differences in the series of samples); a crystallinity was around 40%.

**Figure 7:**
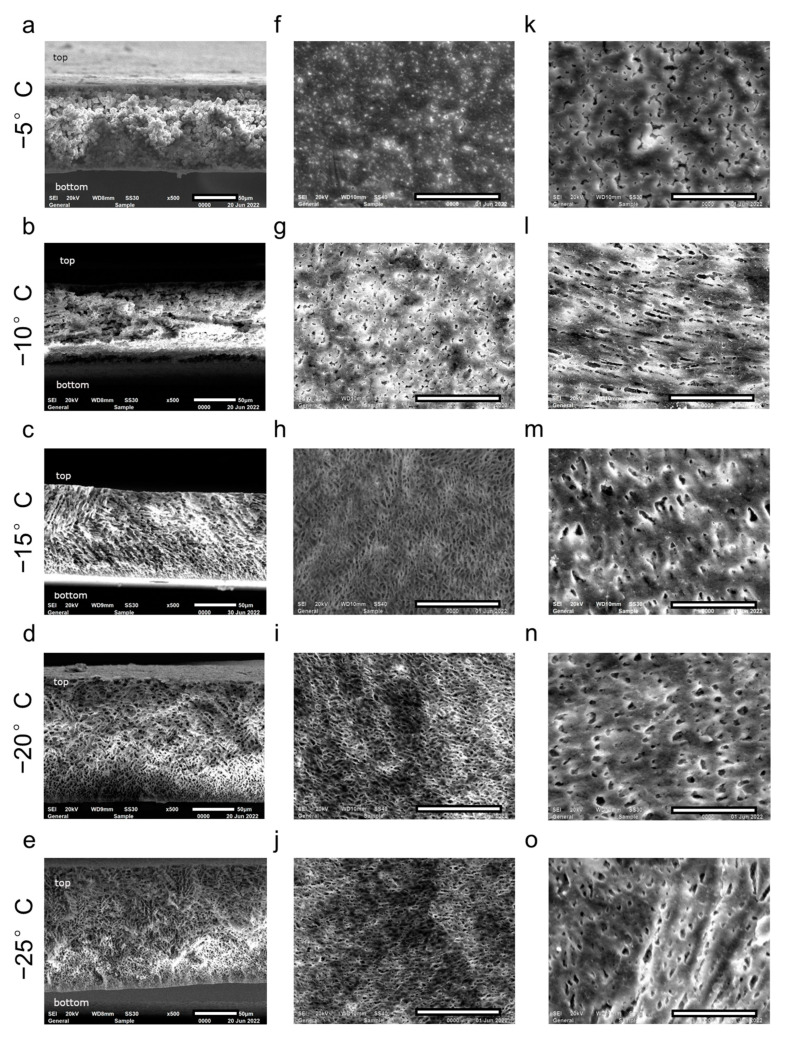
SEM images showing the entire membrane cross-sections (**a**–**e**), magnified the separation surface of the membrane (**f**–**j**) and the top surface of the membrane (**k**–**o**) prepared at different freezing temperatures (the length of the scale bar 50 μm).

The reason for the observed dependencies is the difference in the formed membranes’ structure at varying freezing temperatures, which is shown in Figure 7.

**Figure 8:**
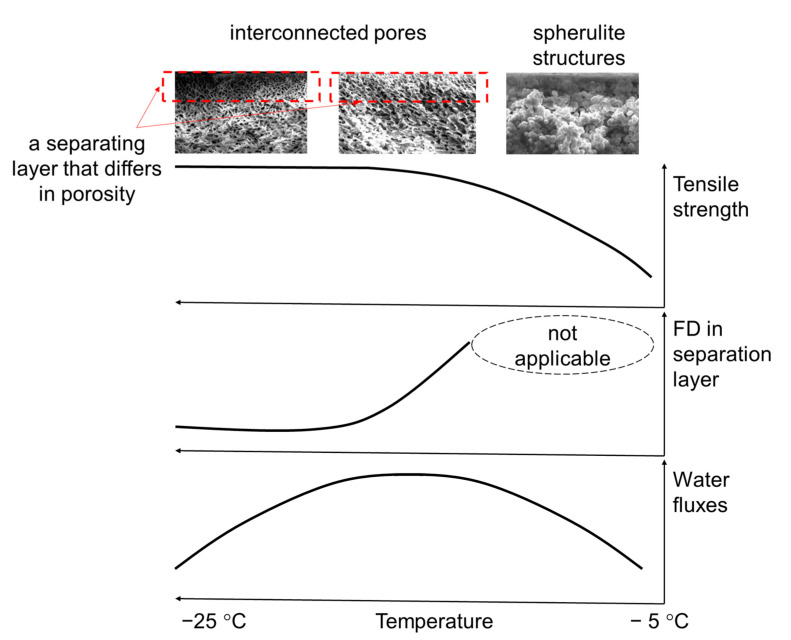
Scheme explaining the correlation between the properties of membranes prepared at different freezing temperatures and their pore structure.

The schematic explanation of the correlation between the properties of membranes prepared at different freezing temperature and its pore structure is shown in Figure 8.

At −5 °C, the membrane has the least strength, which is associated with the formation of spherulite structures (Figure 7), which are more discrete and unevenly integrated into the membrane volume. This trend is generally recognized; the effect of freezing temperature on the physical and mechanical features of the formed porous structures is discussed in detail in [37,38].

As it is shown in Figure 7, with a decrease in the freezing temperature, there is a transition from the PVDF spherulite structures to the formation of a system of interconnected pores (the transition structures for the studied system can be observed at a temperature of −10 °C and partially at −15 °C).

In membranes produced within the −5 to −10 °C range, it is not possible to observe a separating layer that differs in porosity. Therefore, the pore size within the separating layer, according to the results of SEM image processing, has not been evaluated and there are no data for these temperatures in Figure 6. We have assessed only the FD on the separating surface (Figure 6), which is consistent with water fluxes: the membranes produced at −5 °C are characterized by the smallest FD on the separating surface and water flux. Small water fluxes for membranes produced at −5 °C can be explained by the fact that in the process of delamination of the polymer-solvent system, when the temperature changes, the solidification of the phases is delayed and the coalescence (enlargement) of the droplets occurs, which leads to the formation of “blind” pores or their significant heterogeneity in size.

At a freezing temperature of −15 °C, it is visible that a system of interconnected pores has formed during the freezing process. A separating layer (a layer in contact with a glass substrate and a metal plate) having smaller pores can be distinguished, which increases the farther from the separating layer they are (Figure 6 shows the pore size according to the results of SEM image processing with ImageJ).

Further, as the freezing temperature decreases, the interconnected pore system is maintained, while the pore size in the separating layer decreases (Figure 6), the data on water fluxes and pore size of the separating layer according to the results of SEM image processing and FD on the separating surface are consistent with each other. This trend has also been described in [28]. Once the system of interconnected pores has formed, the strength characteristics of the membranes retain the same level (Table 2).

Comparing the SEM images (for the production of membranes, we have used *F2M-E* PVDF (HaloPolymer, LLC, Kirovo-Chepetsk, Russia), MFR 1.2 g/10 min (230 °C/5 kg)), to the data presented in [26,28,36] for membranes produced by freezing at −10 °C from Solef 1008 (MFR 24 g/10 min (230 °C/5 kg), Kynar K-761 (MFR 12–17 g/10 min (230 °C/21.6 kg)), Solef 1015 (MFR 3.7 g/10 min (230 °C/21.6 kg)), differing in molecular weight, it can be noted that the lower the molecular weight of the polymer used for the production of membranes, the lower the spherulite structures to a system of interconnected pores transition temperature is. Such dependence can be explained by the fact that the lower the molecular weight of the polymer, the higher the mobility of its macromolecular chains at a given temperature and the greater the potential for the formation of spherulite formations is.

Thus, the most important factors determining the structure and performance (separation capability) of PVDF membranes produced by freeze-casting are the concentration of the solution, the molecular weight of the polymer, and the freezing temperature. To produce the least pore-size PVDF membranes from a solution in DMSO one needs a polymer of greater molecular weight, an optimal solution concentration (depending on the brand of polymer used), and a low freezing temperature.

## 4. Conclusions

As a versatile freeze-casting technology for pore materials, this technology has wide prospects for PVDF membrane fabrication and is under extensive investigation [26,27,28]. In these comprehensive studies, the fundamental principles of structure formation of fluoro-containing membranes at solvent crystallization under overcooling are considered.

Following this goal, we studied the process of obtaining membranes based on PVDF by freeze-casting in DMSO. Our research was devoted to the influence of technological factors, such as concentration, freezing temperature, and freezing duration, on the membrane microstructure, as well as on some significant membrane properties, such as porosity, tensile strength, and water permeability. Based on the assessment of the porosity and the mechanical characteristics of the obtained membranes, the optimal concentration of PVDF solution in DMSO has been selected as 25 wt.%.

The microstructure of pores formed in membranes during freezing was also studied. The different membrane structures were observed for a variety of freezing temperatures: for membranes produced at temperatures from −5 to −10 °C range. In this range, the spherulitic structures without a separating layer could be observed. At −15 °C there is a system of interconnected pores with separating layers; further, as the freezing temperature decreases, the interconnected pores are obtained. The pore size in the separating layer decreases.

Comparing the SEM images of membranes prepared by freezing from polymers with different molecular weights (according to MFR), it was noted that lowering the molecular weight of the polymer used for membranes production causes the lowering of the spherulite structures to a system of interconnected pores transition temperature is. The observed structure alteration has affected the most important operational parameter of the membranes—the water permeability, which changes along the curve with an extremum accompanying the decrease in freezing temperature. The maximum that occurs at −15 °C was 1100 L/m^2^ × h.

In the development of ideas about the formation of membranes during the crystallization of a solvent that is under supercooling, that is, in a meta-stable state, the authors carried out observations of the spread of crystallized areas in time. It has been established that the emergence and growth of crystallized areas do not occur over the entire contact surface of the solution with the substrate. Crystalline areas originate in local areas of the solution and subsequently spread throughout the volume of the solution, which leads to the formation of contact borders between the crystallized areas. The size of these crystallized areas is about 10–20 mm. Thus, the architecture of membranes obtained by freeze casting can potentially be extremely inhomogeneous: the pore structure in the center of the growing crystallized area will differ from the pore structure at the periphery of such areas, and even more so along the contact borders of the crystallized areas. This inhomogeneity of the membrane structure should lead to a significant scatter of properties over its area.

Research in the field of studying the formation of crystal nuclei, the kinetics of the propagation of crystallized areas, the features of the heterogeneity of the structure of membranes, and the development of approaches to control these processes are planned by the authors in the next stage of this work.

## Figures and Tables

**Table 1:** Conditions for PVDF membranes production.

PVDF-DMSO Solution Concentration, wt%	Inside-the-Freezer Temperature, °C	In-Freezer Time, min
10	−10	120
15 20 25 25 25 25 25	−10 −10 −10 −5 −15 −20 −25	120 120 15, 120 15 15 15 15

**Table 2:** Characteristics of the produced membranes depending on the solution freezing temperature.

Inside-the-Freezer Temperature, °C	Thickness, µm	Porosity, %	Tensile Strength, MPa	$H_{m}$	$\chi_{c}$
–5	135 ± 0.3	66.2 ± 2.6	1.26 ± 0.13	42.10 ± 4.67	40.2 ± 4.4
–10	142 ± 0.4	63.9 ± 3.0	3.56 ± 0.36	41.85 ± 4.73	40.0 ± 4.6
–15	140 ± 0.3	62.5 ± 3.4	3.22 ± 0.32	41.78 ± 2.70	39.9 ± 2.7
–20	156 ± 0.9	60.0 ± 2.1	2.88 ± 0.29	42.60 ± 2.13	40.7 ± 2.2
–25	180 ± 1.5	59.8 ± 2.7	3.09 ± 0.31	44.16 ± 4.48	42.2 ± 4.4

## Supplementary Materials

The following supporting information can be downloaded at: https://www.mdpi.com/article/10.3390/polym14235283/s1, Video S1: Growing of crystallized areas during the membrane formation at −10 °C.
